# Factors Associated With Emergency Department Visits Among Patients Receiving Publicly-Funded Homecare Services: A Retrospective Chart Review From Southern Taiwan Regional Hospital

**DOI:** 10.34172/ijhpm.2023.7377

**Published:** 2023-10-11

**Authors:** Wen-Yi Chiu, Ta-Chuan Yeh, Chia-Chi Yang

**Affiliations:** ^1^Department of Family Medicine, Kaohsiung Armed Forces General Hospital, Kaohsiung, Taiwan.; ^2^The Master Program of Long-Term Care in Aging, College of Nursing, Kaohsiung Medical University, Kaohsiung, Taiwan.; ^3^Department of Psychiatry, Tri-Service General Hospital, National Defense Medical Center, Taipei, Taiwan.; ^4^Center for Long-Term Care Research, Kaohsiung Medical University, Kaohsiung, Taiwan.; ^5^Department of Medical Research, Kaohsiung Medical University Hospital, Kaohsiung, Taiwan.

**Keywords:** Emergency Department Use, Integrated Homecare Services, Retrospective Chart Review, Southern Taiwan

## Abstract

**Background:** The public health strategy of increasing access to comprehensive home or community-based healthcare services and emergency home visits is intent on reducing the overcrowding of emergency departments. However, scientific evidence regarding the association between home-based healthcare services and emergency department uses is surprisingly insufficient and controversial so far. The present retrospective study identified the risk factors for emergency department visits among patients receiving publicly-funded homecare services.

**Methods:** The personal demographic and medical information, caregiver characteristics, and behaviours related to homecare services and emergency department visits from the medical records and structured questionnaires of 108 patients who were recipients of integrated homecare services in a regional hospital in southern Taiwan between January 1, 2020, and December 31, 2020, were collected. After screening the potential predictor variables using the preliminary univariate analyses, the multivariate logistic regression with best subset selection approach was conducted to identify best combination of determinants to predict unplanned emergency department utilizations.

**Results:** Best subset selection regression analysis showed Charlson Comorbidity Index (odds ratio (OR)=1.33, 95% CI=1.05 to 1.70), male caregiver (OR=0.18, 95% CI=0.05 to 0.66), duration of introducing homecare services (OR=0.97, 95% CI=0.95 to 1.00), working experience of dedicated nurses (OR=0.89, 95% CI=0.79 to 0.99) and number of emergency department utilizations within previous past year before enrollment (OR=1.54, 95% CI=1.14 to 2.10) as significant determinants for unplanned emergency department visits.

**Conclusion:** The present evidence may help government agencies propose supportive policies to improve access to integrated homecare resources and promote appropriate care recommendations to reduce unplanned or nonurgent emergency department visits among patients receiving homecare services.

## Background

**Key Messages**

**Implications for policy makers**

- Policy-maker should be aware that provision of continuous and comprehensive integrated homecare model would reduce unnecessary or avoidable visits to the emergency department (ED).
- Patients who did not utilize unplanned emergency medical resources received a longer time in integrated homecare publicly-funded homecare programs.
- The optimal parsimonious five-determinant model, comprising of Charlson Comorbidity Index, caregiver’s sex, duration to introduce homecare services, working experience of dedicated nurses and number of ED visits within previous past year before enrollment could predict unplanned ED utilizations among patients receiving publicly-funded integrated homecare services.

**Implications for the public**

Increased accessibility of comprehensive home or community-based healthcare services and emergency home visits would reduce overcrowding of emergency departments (EDs). Efforts to reduce and prevent unnecessary or avoidable ED visits should be informed by an understanding of the contributions of specific risk factors for ED utilization for patients receiving integrated home care. The current work authenticated that providing a continuous and comprehensive integrated homecare model would potentially reduce unnecessary or avoidable visits to the EDs. The evidence of demographic and clinical determinants of ED utilization may help government agencies propose supportive policies for improved access to integrated homecare resources and promote appropriate care recommendations to reduce unplanned or nonurgent ED visits among patients receiving homecare services.

The number and proportion of the population aged 65 years and above in Taiwan is rapidly rising. According to the population projections report from the National Development Council, Taiwan officially entered the stage of an “aged society,” as Taiwanese people aged 65 years and above accounted for more than 14% of the country’s total population at the end of March 2018. It is estimated that the percentage of people who are 65 years of age or older is projected to reach 20% in 2025, making it a “superaged society.”^1^ More surprising is that it will take only 7 years for Taiwan to advance from the “aged society” stage to the “superaged society” stage. In comparison, 11 years will be needed for Japan, 15 years for the United States, 29 years for France, and 51 years for the United Kingdom. At the same time, the disability rate in the general population in Taiwan has continued to grow, and the disabled population is estimated to increase from more than 750 000 in 2010 to approximately 1 200 000 by 2031.^2^ Therefore, the demand for healthcare services, including medical and long-term care, will inevitably increase. Unfortunately, less or unsuitable access to healthcare resources often results in unmet medical and long-term care needs and fragmented healthcare. It is imperative to adopt a plan of action to introduce more flexible, accessible, continuous and comprehensive patient-centered healthcare services for eligible elderly or disabled individuals.

 In response to the challenges of unmet healthcare service needs and to construct a complete local home healthcare system, publicly-financed integrated homecare launched by the National Health Insurance Administration, Ministry of Health and Welfare, Taiwan, in 2016 has been proposed to strengthen the connection between medical treatments and healthcare resources and improve the continuity of healthcare delivery. The Integrated Homecare services, which encompass the stages of “homecare,” “intensive homecare,” and “hospice care,” are delivered through contracted homecare teams composed of regulated health professionals, mainly family physicians and registered nurses. When advanced medical and healthcare services are necessary, dentists, Chinese medicine practitioners, pharmacists, respiratory therapists or other medical personnel are further linked. Under the regulation of Integrated Homecare, if a patient living at home has a definite need for medical or nursing services, but it is difficult to reach out the essential services because of limited self-care ability with a score of less than 60 for the Barthel activities of daily living (ADL) index or specific illnesses conditions, the patient would be eligible to apply the reimbursed homecare items, including physician and nursing homecare, clinical diagnosis and treatment, the provision of medical supplies and general nursing care and laboratory tests, etc. Intensive homecare covers the reimbursed homecare items and additional special skilled nursing services, such as replacing nasogastric tubes, tracheostomy tubes, Foley catheters and wound care. Moreover, the hospice care stage refers to patients qualified for the homecare reimbursement receiving the hospice and palliative care under terminal illness or end-stage disease.^3^ According to the 2019 Health and Welfare Report published by the Ministry of Health and Welfare, Taiwan, more than 1200 contracted medical service institutions form professional integrated homecare teams, providing care for over 49 000 qualifying individuals in 2018.^4^ Since the demand for healthcare has skyrocketed, substantial growth in homecare services is expected in the foreseeable future.

 Although the public health strategy of increased accessibility of comprehensive home or community-based healthcare services and emergency home visits have been commonly adopted to reduce overcrowding of emergency departments (EDs),^5-13^ scientific evidence on the association between home-based healthcare services and ED usage is surprisingly insufficient and controversial. Provision of integrated homecare services has been shown to be beneficial for reducing inappropriate ED visits,^10,12,13^ whereas contrary argument exhibits unplanned or nonurgent ED visits often occur among patients receiving homecare services.^14,15^ Given that reducing unnecessary ED visits is widely thought as an important goal of all primary care and specialty practices and a challenging and contentious issue for policy-makers, efforts to reduce and prevent unnecessary or avoidable ED visits should be informed by an understanding of the contributions of specific risk factors for ED utilizations in this population.

 Therefore, the objective of this study was to identify the risk factors for ED visits among patients receiving publicly-funded homecare services. As the first step in this work, the available independent measures were reviewed to explore personal demographic and medical information, caregiver characteristics and behaviors related to homecare services and ED visits that would or would not be related to the utilizations of ED. Next, these candidate independent measures were reduced into a more economical set of risk factors correlated with ED utilizations.

## Methods

###  Study Design and Setting 

 This single-center, retrospective observational study was based on a review of charts from patients receiving the publicly-financed integrated homecare programs in Kaohsiung Armed Forces General Hospital, which is a regional hospital located in southern Taiwan and is a dedicated institution coordinating and offering appropriate healthcare services for eligible elderly or disable individuals. Moreover, both the study protocol and the process of retrospective chart review were approved by the institutional review board of the Kaohsiung Armed Forces General Hospital, Taiwan (KAFGHIRB 109-056). Because of the nature of this retrospective study and data de-identification, the requirement for informed consent was waived.

###  Participants

 Patients eligible for reimbursement under integrated homecare programs between January 1, 2020 and December 31, 2020, and over the age of 18 years were initially recruited into the retrospective cohort study. If patients were receiving hospice care services, did not complete the assessments regarding the personal demographic information, health status, physical functions, medications, nutrition, conscious levels, and comorbidities etc, or did not receive more than three months of homecare services, they were removed from the current study. The patients who had visited the ED once during the study period were registered in the case group.

###  Data Sources

 The whole data analyzed in the current retrospective study were mainly retrieved by from two major information sources. One is the hospital information system database, which is the official digitized records for patients’ socio-demographics, medical information and details on homecare services and ED utilizations. Moreover, as patients were initially enrolled in the publicly-financed integrated homecare programs, each of them formally received a series of standardized assessments for comprehensively evaluating health status, physical functions, medications, nutrition, conscious levels, and comorbidities etc, which were also documented in the hospital information system, and updated quarter-yearly or if there is a significant change in clinical status. Caregiver and nurse characteristics were obtained from another information source, the structured questionnaires. Theses collected data identifying beneficiaries, caregivers and dedicated nurses were encrypted to ensure privacy and then constructed multiple-linked population-based health administrative datasets.

###  Variables 

 The variables used for analysis in the present retrospective study were mainly comprised of the patients’ demographics and medical information, caregiver and nurse characteristics, and behaviors on homecare service and ED visits. Apart from sex and age, patients’ medical information included nasogastric intubation, Foley catheter insertion and skin pressure ulcer, bedridden status, number of prescribed drugs and scores on the Glasgow Coma Scale, Barthel ADL Index, and Charlson Comorbidity Index. Caregiver and nurse characteristics involved full or part-time care pattern, caregiver’s age, sex, nationality, and working experience of dedicated nurses. Finally, behaviors related to homecare services and ED visits were number of ED utilizations within the period of data collection, duration of introducing homecare services, previous one-year experience with ED utilizations. Of these variables, patients’ demographics and medical information, behaviors related to homecare services, and ED utilizations were retrieved from the hospital information system databases. Caregiver and nurse characteristics were further gathered through structured questionnaires from patients, family members, caregivers and dedicated nurses. All charts in the current work were reviewed and appropriateness by two of the co-authors (WYC and CCY). In unclear cases, the other co-author was consulted (TCY).

###  Statistical Analysis

 Descriptive statistics were initially used to feature the personal demographic and medical information and summarize the characteristics of caregivers and previous behaviors regarding homecare services and ED visits for all participants. Preliminary univariate analyses, including the independent *t *test or Mann–Whitney U test for continuous variables and chi-square analysis or Fisher’s exact test for categorical variables, were conducted to define the potential determinants of ED utilizations. The potential determinants that achieved a *P* value ≤.2 in the preliminary univariate analyses were further entered into the multivariate logistic regression model to identify the explanatory risk factors associated with ED utilization. To find the subset of potential determinants that best predict the ED utilizations and combat the pervasive overfitting or underfitting problems, the best subset selection approach was employed.^16^ Subsequently, the parsimonious multivariate logistic regression model was selected based on with the minimum Akaike information criterion (AIC).^17^ All analyses in the current work were conducted using R version 4.2.3 software and the threshold for statistical significance for all analyses was* P*< .05.

## Results

 A total of 122 patients were identified and 12 of them did not receive more than three months of integrated homecare services. Of the remining 110 eligible participants, 2 patients were found to have insufficient clinical documentation to be included in the analysis. The cohort study finally contained 108 patients. A detailed description of the study flowchart can be found in Figure. Patients had a mean age of 80.8 (±14.1) years and were almost balanced between males (53.7%) and females (46.3%). There were 68 (63.0%) and 63 (58.3%) patients who underwent nasogastric intubation and Foley catheter insertion, respectively. The mean scores were 9.4 (±14.9) for the Barthel ADL Index and 7.1 (±2.1) for the Charlson Comorbidity Index. The average duration of receiving publicly-funded integrated homecare programs was 24.4 (±21.9) months. Of the 108 patients, 65 patients (60.2%) used ED services within the data collection period, with a mean number of 2.1 (±1.7) for ED utilizations. Other characteristics of patients contributing to the analysis can be found in Table 1.

**Figure:**
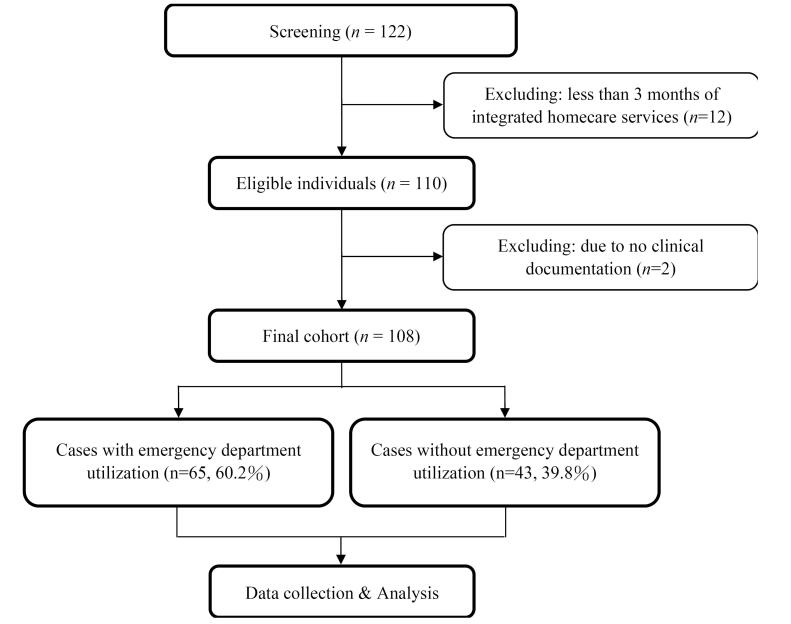
Flowchart for Participant Screening and Enrollment.

**Table 1:** Personal Characteristics and Univariate Analyses of Collected Variables for All Participants

	**Total**	**Patients With ED Utilizations (n = 65)**	**Patients Without ED Utilizations (n = 43)**	***P *****Value**^e^
Basic demographic and medical information				
Mean age (y)	80.8 ± 14.1	81.5 ± 12.2	79.7 ± 16.6	.506^b^
Male, n (%)	58 (53.7)	36 (55.4)	22 (51.2)	.410^c^
Welfare receipt	80 (74.1)	51 (78.5)	29 (67.4)	.201^c^
Glasgow Coma Scale	12.4 ± 3.1	12.3 ± 3.2	12.7 ± 2.9	.452^b^
Nasogastric intubation, No. (%)	68 (63.0)	46 (70.8)	22 (51.2)	**.032**^c^
Foley catheter insertion, No. (%)	63 (58.3)	35 (53.8)	28 (65.1)	.168^c^
Skin pressure ulcer, No. (%)	23 (21.3)	19 (29.2)	4 (9.3)	**.011**^d^
Bedridden, No. (%)	41 (38.0)	22 (33.8)	19 (44.2)	.189^c^
Barthel ADL index	9.4 ± 15.0	8.5 ± 15.1	10.6 ± 4.57	.487^b^
Charlson Comorbidity Index	7.1 ± 2.1	7.4 ± 2.1	6.5 ± 2.0	**.030**^b^
Polypharmacy^a^	87 (80.6)	54 (83.1)	23 (53.5)	.416^c^
No. of prescribed drugs	6.7 ± 2.9	7.1 ± 2.9	6.1 ± 2.9	.096^b^
Caregiver/Nurse characteristics				
Full-time caregiver, No. (%)	80 (74.1)	50 (76.9)	30 (69.8)	.271^c^
Age ≥65 years, No. (%)	22 (20.4)	13 (20)	9 (20.9)	.546^c^
Male caregiver, No. (%)	19 (17.6)	9 (13.8)	10 (23.3)	.159^c^
Foreign nationality, No. (%)	46 (42.6)	29 (44.6)	17 (39.5)	.374^c^
Mean year of working experience for dedicated nurse (y)	10.2 ± 4.2	9.7 ± 4.1	10.9 ± 4.3	.148^b^
Behaviors on home care service and ED visit				
No. of ED utilizations within the period of data collection (min-max)	N/A	2.1 ± 1.7 (1-9)	N/A	N/A
Mean duration of receiving home care services (mon)	24.4 ± 21.9	20.3 ± 17.3	30.6 ± 26.4	**.016**^b^
No. of ED utilizations over the past year	1.2 ± 1.6	2.4 ± 2.5	0.9 ± 1.1	**<.001**^b^

Abbreviations: ADl, activities of daily living; ED, emergency department; N/A, not applicable. ^a^Polypharmacy was defined as regular use of at least five prescribed drugs. ^b^*P* values comparing patients with ED utilizations and those without ED utilizations within the data collection period were obtained from independent *t* test. ^c^*P* values comparing patients with ED utilizations and those without ED utilizations within the data collection period were obtained from chi-square analysis. ^d^*P* values comparing patients with ED utilizations and those without ED utilizations within the data collection period were obtained from Fisher’s exact test. ^e^A *P *value of less than.05 was considered significant.

 The preliminary univariate analyses demonstrated that a higher proportion of participants with ED utilizations presented with nasogastric intubation (70.8% vs. 51.2%, *P*= .032) or skin pressure ulcers (29.2% vs. 9.3%, *P*= .011) compared with those without ED utilizations. In addition, the mean score for the Charlson Comorbidity Index and the number of ED visits over the year before the index period were significantly higher in the case group (*P*< .05). The patients who did not utilize unplanned emergency medical resources during the study period received a longer duration of homecare services, with a mean length of 30.6 (±26.4) months (*P*= .016). As achieving a *P* ≤.2 in the preliminary univariate analyses, a total of 10 candidate determinants, including nasogastric intubation, Foley catheter insertion, skin pressure ulcer, bedridden status, Charlson Comorbidity Index, number of prescribed drugs, caregiver’s sex, duration of introducing homecare services, working experience of dedicated nurses and previous one-year experience with ED utilizations were included into the multivariate logistic regression. Finally, the best subset selection analysis directly yielded the optimal five-determinant model which was selected with the minimum AIC value of 123.41, revealing Charlson Comorbidity Index, caregiver’s sex, duration of introducing homecare services, working experience of dedicated nurses, and number of ED utilizations within previous past year before enrollment as independent risk factors for ED visits (Table 2). Table 3 summarizes the details regarding the risk factors related to ED utilizations based on the best subset selection analysis. The parsimonious multivariate logistic regression model satisfied the omnibus test of model coefficients (*P*< .001) and the Hosmer–Lemeshow test (*P*= .521), indicating a good fit model. Patients having higher scores for the Charlson Comorbidity index (odds ratio [OR] = 1.33, 95% confidence interval [CI] = 1.05 to 1.70) and prior experience with ED utilizations in the past one year (OR = 1.54, 95% CI = 1.14 to 2.10) had significantly higher risk for unplanned ED utilizations during the data collection timeframe. Moreover, receiving homecare services offered by male caregiver (OR = 0.18, 95% CI = 0.05 to 0.66), longer duration of introducing home care services (OR = 0.97, 95% CI = 0.95 to 1.00) and prolonged working experience of dedicated nurse (OR = 0.89, 95% CI = 0.79 to 0.99) were significantly associated with a lower risk for ED visits.

**Table 2:** Best Subset Selection Approach for Screening Best Combination of Predictor Variables

	**Number of Variables Included**									
	**1**	**2**	**3**	**4**	**5**	**6**	**7**	**8**	**9**	**10**
Variables^a^										
Nasogastric intubation						✓		✓	✓	✓
Foley catheter insertion							✓	✓	✓	✓
Skin pressure ulcer		✓	✓				✓	✓	✓	✓
Bedridden									✓	✓
Charlson Comorbidity Index				✓	✓	✓	✓	✓		✓
No. of prescribed drugs									✓	✓
Male caregiver				✓	✓	✓	✓	✓	✓	✓
Mean year of working experience for dedicated nurse			✓		✓	✓	✓	✓	✓	✓
Mean duration of introducing home care services				✓	✓	✓	✓	✓	✓	✓
No. of ED utilizations over the past year	✓	✓	✓	✓	✓	✓	✓	✓	✓	✓
AIC	132.54	130.12	128.16	126.37	123.41	123.52	123.67	125.02	126.65	128.46

Abbreviations: ED, emergency department; AIC, Akaike information criterion. ^a^Variables that achieved a *P* ≤ .2 in the preliminary univariate analyses were entered into the multivariate logistic regression analyses with best subset selection approach to determent the parsimonious model.

**Table 3:** Multivariate Logistic Regression Analysis of Factors That Can Independently Predict Uses of Emergency Medical Resources Within the Data Collection Period

	**OR**	**95% CI**	***P***** Value**
Charlson Comorbidity Index	1.33	1.05-1.70	**.021**
Male caregiver	0.18	0.05-0.66	**.010**
Mean duration of introducing home care services (mon)	0.97	0.95-1.00	**.015**
Mean year of working experience of dedicated nurse (y)	0.89	0.79-0.99	**.030**
No. of ED utilizations over the past year	1.54	1.14-2.10	**.006**

Abbreviations: OR, odds ratio; ED, emergency department; CI, confidence interval. *Note: *Omnibus test of model coefficients: *P* < .001; Hosmer–Lemeshow test: *P* = .521.

## Discussion

 As the explosive growth in healthcare demand for healthcare services, provision of integrated home and community-based care services is assumed to ensure continuity of healthcare delivery, reduce unnecessary or avoidable visits to the hospital or ED.^5-11^ Nonetheless, concerns about the appropriateness of ED utilizations among the population receiving homecare services still persist.^18^ In the current study, we aimed to examine personal demographic and medical information, caregiver characteristics and behaviors related to homecare services and ED visits to explore the risk factors for ED visits among patients receiving publicly-funded homecare services. The preliminary univariate analyses showed that the presences of nasogastric intubations and skin pressure ulcers, more severe comorbid condition, a shorter duration for receiving homecare services and high use of ED services within one year prior to data collection were associated with unplanned ED utilizations among patients receiving publicly-funded integrated homecare services. Notably, to the authors’ knowledge, our study is the first attempt to leverage best subset selection regression approach to stratify the risk of utilizing ED sources among patients receiving publicly-funded homecare services. Selecting the variables with *P*≤ .2 in the preliminary univariate analyses, the satisfactory parsimonious five-determinant model was registered, disclosing Charlson Comorbidity Index, caregiver’s sex, duration of introducing homecare services, working experience of dedicated nurses and number of prior ED utilizations in the past one year to be the most predictive of the likelihood of unplanned ED utilizations. Considering these results, the risk stratification prediction model may be feasible for early identifying recipients receiving publicly-funded integrated homecare services at risk for ED visits.

 It is generally acknowledged that Charlson Comorbidity Index is the commonly-used gold-standard measure to assess comorbidity in clinical scenarios^19^ and a proxy for multimorbidity and morbidity burden in primary care and community settings.^20^ Individuals with higher levels of comorbidities are more susceptible to sudden health deteriorations.^21^ As might be expected, our results from the univariate analyses revealed that patients receiving homecare services with the experience of ED visits had higher scores for the Charlson Comorbidity index. The findings from the best subset selection regression analysis conducted in the present work parallel other previous investigations that substantially confirmed comorbidity being as an independent predictive factor for hospital mortality, ED utilization and recidivism for elders.^22,23^

 Another relevant indicator of unplanned ED visits is represented by the duration of provision of healthcare services. Our study not only found that the patients who did not utilize unplanned emergency medical resources within the observation period received a longer time in integrated homecare programs but also displayed duration of receiving home care services could be used to determine the likelihood of unplanned ED utilizations. Consistent with our finding, a before-after retrospective cohort study conducted in Vancouver, Canada, in a sample of 246 infirm participants aged over 55 years, showed that ED visit rates after enrollment in integrated home-based primary cares did not significantly decrease but tended toward stabilization.^24^ Another time series analyses in Italy, consisting of 39 822 recipients receiving integrated home cares also reported 45%, 17%, and 64% reduction in ED visits after introducing short, intermediate and long duration of integrated home cares, respectively.^10^ Accordingly, continuously accessing integrated homecare services could be beneficial for risk mitigation of unplanned ED utilizations.

 Moreover, our study identified the characteristics of caregiver or dedicated nurse, including caregiver’s sex and working experience as independent risk factors unplanned or nonurgent ED visits among patients receiving homecare services. Previous studies have reported that female caregiver perceive caregiving as more burdensome because of socialization and role expectations^25^ and different coping strategies for caregiving situation,^26,27^ which may account for the lower risk of unplanned or nonurgent ED visits among patients receiving homecare services provided by male caregiver. On the other hand, a nurse-centric survey from the National Database of Nursing Quality Indicators^TM^ (NDNQI)^®^ in 2006 concluded the relationship between nurse experience and quality of care.^28^ In line with this, our multivariate logistic regression results presented that for every increase of one year in average working experience of dedicated nurse, the risk of unexpectedly using ED recourses was lowered by 1.1% lower.

 Our result also supports the common notion, indicating that past use of healthcare services is one of the important variables influencing subsequent healthcare utilizations among the geriatric population.^29-31^ The finding of the present work based on the best subset selection regression analysis demonstrated that a higher number of past ED visits was predictive of the likelihood of ED utilization during the index period. In other words, patients with one year of previous experience with ED utilizations were more likely to visit emergency services unexpectedly. Research from a medical center in Taipei, Taiwan, has also reported that multiple previous ED visits appeared to be one of the risk factors for readmissions or ED utilization among patients receiving home healthcare.^32^ Analogous findings were obtained from Franchi et al, who noted that a higher number of past ED visits was another predictor significantly associated with frequent ED use, showing that seniors with four or more department visits had a 30-fold higher risk of having the same number of ED utilization in the subsequent year.^30^

 Of note, empirical data have disclosed that nasogastric intubation-induced aspiration would be a potentially serious complication and a significant risk factor for unplanned emergency readmission.^33,34^ The present work found that more than 60% of participants (68/108) receiving publicly-funded integrated homecare services had nasogastric intubations and further manifested significantly higher proportion of patients having nasogastric intubation visited the ED than those who did not. Since the demand for nasogastric intubation in disabled older homecare residents in Taiwan continues to grow,^35,36^ effective education strategies are urgently needed to manage accurate nasogastric tube feeding and prevent unplanned or accidental extubation and ED utilization for this reason.

 The present findings should be read while considering some embedded limitations; first, given that our single-center, retrospective design just collected the specific targets, only patients who received homecare services integrated by a dedicated regional hospital in southern Taiwan were recruited for this study. The relatively small sample size and the sample specificity, therefore could lack the representativity of whole home healthcare systems in Taiwan and the present work may not be suitable for extrapolation to other home healthcare systems. Second, published evidence indicated sharp increase in the number of patients who utilized ED or acute healthcare recourses as approaching death.^37,38^ As we limited the retrospective cohort study to patients receiving more than three months of publicly-funded integrated homecare services within the observation period, patient facing a rapid deterioration in health with imminent death in an emergency medicine setting and receiving less than three months of homecare services were removed from the current database. Our inability to consider another important clinical issue regarding imminent death in an emergency medicine setting might probably bias the findings in unpredictable ways. Additionally, approximately 70% (80/108) of the participants of this study were eligible for social welfare assistance, and further effort is needed to highlight whether governmental medical subsidies would influence the behaviors in seeking ED sources. Finally, we reviewed charts of patients enrolled in a regional hospital’s publicly-financed integrated homecare programs. Records regarding the use of EDs in other hospitals were unavailable, and the results would likely be underestimated.

## Conclusion

 In addition to the presences of nasogastric intubations and skin pressure ulcers, more severe comorbid condition, a shorter duration for receiving homecare services and high use of ED services within one year prior to data collection were highlighted to be associated with unplanned ED utilizations among patients receiving publicly-funded integrated homecare services, specially, we identified the optimal parsimonious five-determinant model, disclosing Charlson Comorbidity Index, caregiver’s sex, duration of introducing homecare services, working experience of dedicated nurses, and number of prior ED utilizations in the past one year to be the most predictive of the likelihood of unplanned ED utilizations among patients receiving publicly-funded integrated homecare services. The risk stratification-based prediction model could be incorporated into hospital information system for early warning patients at risk for ED visits. The evidence of demographic and clinical determinants of ED utilizations may also help government agencies propose supportive policies for improved access to integrated homecare resources and promote appropriate care recommendations to reduce unplanned or nonurgent ED visits among patients receiving homecare services.

## Ethical issues

 Ethical approval was obtained by the institutional review board of the Kaohsiung Armed Forces General Hospital, Taiwan (KAFGHIRB 109-056).

## Competing interests

 Authors declare that they have no competing interests.

## Disclaimer

 The manuscript has never been published before in any other journal and will not be submitted to another journal during the period it is under review or after it is accepted by the Journal. The authors agree to follow the Journal’s submission instructions.

## Funding

 This work was supported by Kaohsiung Armed Forces General Hospital, Taiwan and the National Science and Technology Council, R.O.C. (Taiwan) (MOST 111-2221-E-037-003-MY2).
